# Modified Polymeric Nanoparticles Exert In Vitro Antimicrobial Activity Against Oral Bacteria

**DOI:** 10.3390/ma11061013

**Published:** 2018-06-14

**Authors:** Manuel Toledano-Osorio, Jegdish P. Babu, Raquel Osorio, Antonio L. Medina-Castillo, Franklin García-Godoy, Manuel Toledano

**Affiliations:** 1 Dental School, University of Granada, Campus de Cartuja s/n, 18071 Granada, Spain; toledano@correo.ugr.es (M.T.-O.); toledano@ugr.es (M.T.); 2 College of Dentistry, University of Tennessee Health Science Center, 875 Union Avenue, Memphis, TN 381632110, USA; jbabu@uthsc.edu (J.P.B.); fgarciagodoy@gmail.com (F.G.-G.); 3 NanoMyP, Spin-Off Enterprise from University of Granada, Edificio BIC-Granada, Av. Innovación 1, Armilla, 18016 Granada, Spain; amedina@nanomyp.com

**Keywords:** antibacterial, calcium, doxycycline, nanoparticles, zinc

## Abstract

Polymeric nanoparticles were modified to exert antimicrobial activity against oral bacteria. Nanoparticles were loaded with calcium, zinc and doxycycline. Ions and doxycycline release were measured by inductively coupled plasma optical emission spectrometer and high performance liquid chromatography. *Porphyromonas gingivalis*, *Lactobacillus lactis*, *Streptoccocus mutans*, *gordonii* and *sobrinus* were grown and the number of bacteria was determined by optical density. Nanoparticles were suspended in phosphate-buffered saline (PBS) at 10, 1 and 0.1 mg/mL and incubated with 1.0 mL of each bacterial suspension for 3, 12, and 24 h. The bacterial viability was assessed by determining their ability to cleave the tetrazolium salt to a formazan dye. Data were analyzed by ANOVA and Scheffe’s F (*p* < 0.05). Doxycycline doping efficacy was 70%. A burst liberation effect was produced during the first 7 days. After 21 days, a sustained release above 6 µg/mL, was observed. Calcium and zinc liberation were about 1 and 0.02 µg/mL respectively. The most effective antibacterial material was found to be the Dox-Nanoparticles (60% to 99% reduction) followed by Ca-Nanoparticles or Zn-Nanoparticles (30% to 70% reduction) and finally the non-doped nanoparticles (7% to 35% reduction). *P. gingivalis*, *S. mutans* and *L. lactis* were the most susceptible bacteria, being *S. gordonii* and *S. sobrinus* the most resistant to the tested nanoparticles.

## 1. Introduction

Bacteria are the main cause of prevalent oral diseases as caries and periodontitis. Oral administration of antibacterial agents presents an important limitation, as it is accessing the dentin interface, the radicular canal or the subgingival pockets where these bacteria grow. In these cases, benefits of local versus systemic delivery routes are clear [1]. This work explores the design of nanoparticles (NPs) that locally administered will exert therapeutic antibacterial properties against oral bacteria. 

Resin-based restorative materials are commonly employed in clinical treatments to seal the interfaces and as a result, bonding to dentin is challenging [2]. Gaps at these bonded interfaces lead to microleakage, which also facilitate the invasion of cariogenic pathogens to cause secondary caries infections [2,3]. Therefore, in order to minimize the incidence of secondary caries, it would be desirable the existence of an antibacterial agent able to inhibit cariogenic pathogens at the dentin interface [4]. For this purpose, studying antibacterial effects against *Streptococcus mutans* (*Sm*), *Streptococcus gordonii* (*Sg*), *Streptococcus sobrinus* (*Ss*), and *Lactobacillus lactis* (*Ll*) have been recommended [3].

In endodontic treatment, elimination of bacteria in the root canal system, is a major challenge. Microorganisms remained after canal treatment will impair periapical healing and will facilitate developing apical lesions [5]. *Porphyromonas gingivalis* (*Pg*) is a major etiologic agent not only in the recurrent infections after endodontic treatment [6], but also in the development and progression of periapical lesions and periodontitis [7]. Biomaterials design leading to minimize the incidence of persistent or recurrent infections of the root canal system and apical periodontitis would also be desirable [8]. It is important to note that the biosafety of sodium hypochlorite during root canal treatment has been recently questioned [9].

Endogenous matrix metalloproteinases (MMPs) are interstitial collagenases present in radicular dentin, periodontal tissue and periapical bone [10,11,12,13,14]. MMPs have been related to chronic inflammation processes and abscesses at apical level [10,12]. Then MMPs inhibition will improve the prognosis of endodontic treatments [11]. Moreover, if resin-based materials are used for dentin bonding and tooth restoring, collagen degradation by MMPs will occur at the dentin interface jeopardizing restorations longevity [15]. When bonding to dentin in restorative dentistry, if dentin is infiltrated by MMP inhibitors, crystallite-sparse collagen fibrils of the scaffold could be protected from degradation facilitating further remineralization [2,16,17]. Metal nanoparticles (i.e., silver, gold, or zinc oxide…) have been previously introduced in restorative dentistry, mainly due to their antibacterial or MMPs inhibition properties [18,19,20]. 

Novel polymeric nanoparticles (NPs), about 100 nm in diameter, have been synthetized and previously tested at the resin-dentin bonded interface [17,21]. NPs have been shown to inhibit dentin MMPs collagen degradation [22], and to facilitate mineral growth at the interface without impairing bond strength [21,22]. Sequences of anionic carboxylate (i.e., COO^−^) are along the backbone of the polymeric NPs. These functional groups permit the possibility of calcium and zinc quelation (1 µg Ca/mg NPs and 2.2 µg Zn/mg NPs) [23]. Cationic metals, loaded onto particles surfaces, if released, may provide for antimicrobial activity. Both metal cations have been demonstrated to have significant antibacterial effects [24,25]. Moreover, when NPs are larger than 10 nm, they do not penetrate bacteria membranes and are thought to exert further antimicrobial effects through accumulation on cell membranes [26]. At this stage, the bacterial membrane permeability may become compromised rendering the cell unable to regulate transport through it, and eventually causing cell death [27]. Doxycycline hyclate is also an antibacterial [28] and potent MMPs inhibitor [29] that is proposed to be immobilized on presented NPs.

Proposed NPs may be employed in three different dental clinical applications: (1) during the dentin bonding process, to exert antibacterial activity at the resin–dentin bonded interface [17,21]; (2) inside the radicular canal, during the endodontic treatment, to facilitate bacterial elimination; and (3) at the periodontal pocket, onto the cementum surface, to directly exert antibacterial activity [23].

Thus, the purpose of this *in vitro* study was to design and synthetize NPs doped with calcium/zinc ions or with immobilized doxycycline able to exert antibacterial activity against *Sm*, *Sg*, *Ss*, *Ll* and *Pg*. The null hypotheses to be tested are that: (1) Calcium, zinc and doxycycline are not liberated from NPs, and (2) NPs, calcium, zinc and doxycycline doped NPs do not affect bacterial viability. 

## 2. Results

*1. Loading efficacy and release of doxycycline hyclate from NPs:* The amount of doxycycline in the aqueous solution before NPs immersion was 1333 µg/mL (per mg of NPs). In the supernatant, after NPs immersion, doxycycline concentration was 399.5 µg/mL (per mg of NPs). Loading efficacy was around 70%. Mean and standard deviation of doxycycline liberation (µg/mL) and cumulative liberation (%), per 10 mg of NPs at each time point are presented in Table 1. Doxycycline liberation was 106 µg/mL (per mg of NPs) at 12 h. A burst effect with rapid doxycycline liberation was observed from 12 h until the first week of storage. After 7 days, the antibiotic release was above 20 µg/mL (per mg of NPs). After 21 days, doxycycline liberation was stably sustained, being 8 and 6 µg/mL (per mg of NPs) at 21 and 28 days, respectively. After 24 h, a 57% of the immobilized doxycycline was liberated, and after 7 days and 28 days, 72% and 80% of loaded antibiotic was respectively released (Table 1).

*2. Calcium and zinc liberation from NPs:* Mean and standard deviation of Ca^2+^ and Zn^2+^ liberation (µg) and cumulative liberation (%), per 10 mg of NPs at each time point are presented in Table 1. Calcium liberation ranged from 0.856 to 1.007 µg (per 10 mg of NPs) during the first week. This calcium release was doubled after 21 d, being around 2 µg (per 10 mg of NPs). Zn-NPs maintained a sustained zinc liberation that ranged from 0.021 to 0.025 µg (per 10 mg of NPs) between 12 to 21 days. A double fold increase was observed at day 28, when 0.044 µg were released per each 10 mg of NPs.

**Figure 1:**
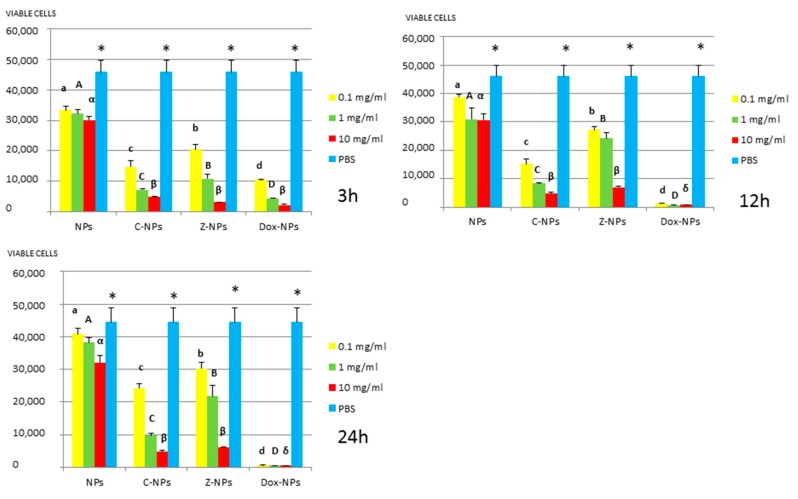
*P. gingivalis* survival (number of viable cells) after 3 h, 12 h, and 24 h of different concentration NPs exposure. Same letter or symbol indicates no significant difference of viable bacteria between different NPs concentrations (*p* < 0.05).

**Figure 2:**
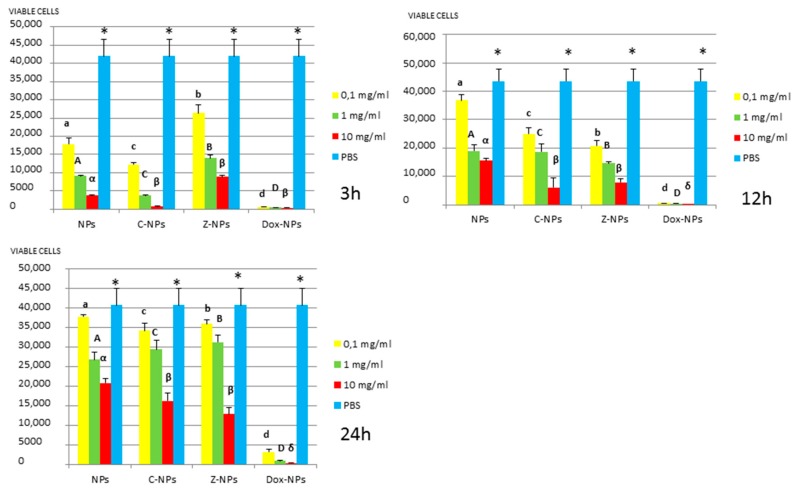
*S. mutans* survival (number of viable cells) after 3 h, 12 h, and 24 h of different concentration NPs exposure. Same letter or symbol indicates no significant difference of viable bacteria between different NPs concentrations (*p* < 0.05).

**Figure 3:**
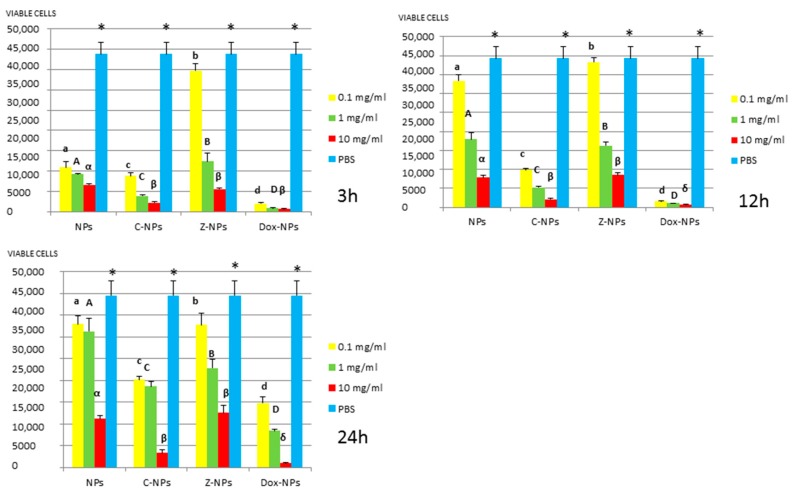
*L. lactis* survival (number of viable cells) after 3 h, 12 h, and 24 h of different concentration NPs exposure. Same letter or symbol indicates no significant difference of viable bacteria between different NPs concentrations (*p* < 0.05).

**Figure 4:**
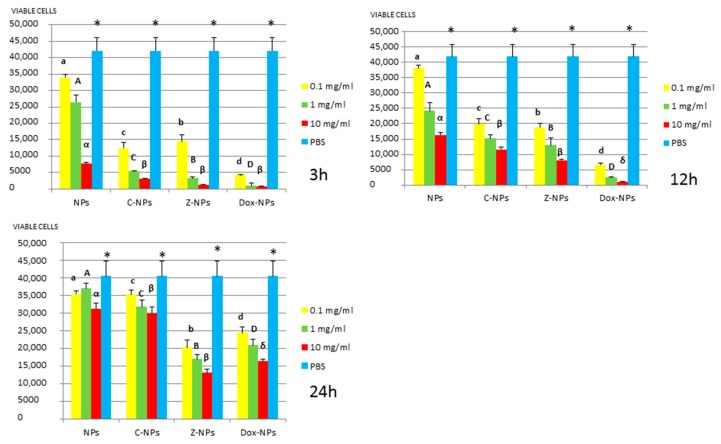
*S. gordonii* survival (number of viable cells) after 3 h, 12 h, and 24 h of different concentration NPs exposure. Same letter or symbol indicates no significant difference of viable bacteria between different NPs concentrations (*p* < 0.05).

**Figure 5:**
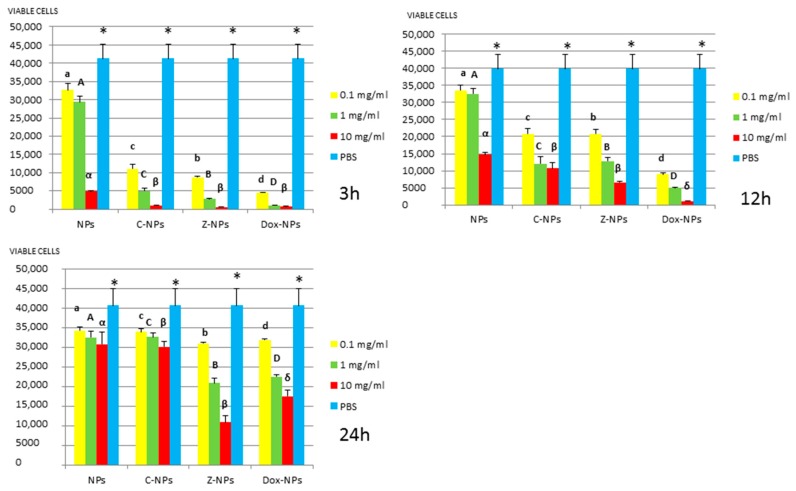
*S. sobrinus* survival (number of viable cells) after 3 h, 12 h, and 24 h of different concentration NPs exposure. Same letter or symbol indicates no significant difference of viable bacteria between different NPs concentrations (*p* < 0.05).

*3. MTT assay:* Mean and standard deviations of the different bacteria survival values expressed as number of viable cells after 3, 12 and 24 h of exposure to the distinct NPs and control PBS are shown in Figure 1, Figure 2, Figure 3, Figure 4 and Figure 5.

In general, all tested NPs affected the viability of bacterial suspension. The most effective were the Dox-NPs followed by Ca-NPs or Zn-NPs and finally non-doped NPs that attained the most variable and least reduction in bacterial survival (8% to 70% after 24 h).

The viability of tested bacteria following the incubation with NPs depends on the type of NPs. The two bacteria, *S. gordonii* and *S. sobrinus* were found to be the most resistant to the tested NPs. After 24 h, they were only affected by Zn-NPs (70% reduction in bacterial viability) and by Dox-NPs (60% reduction). For *P. gingivalis*, *S. mutans* and *L. lactis*, Dox-NPs reduced the bacterial viability by 60% to 99%, after 24 h depending on the concentration of doxycycline. Meanwhile the reduction in bacterial viability were from 20% to 60% for *S. gordonii* and *S. sobrinus*. The *P. gingivalis*, *L. lactis* and *S. mutans* Dox-NPs effect was not variable during the time of the study. Only in the cases of *S. gordonii* and *S. sobrinus* cultures a drop in Dox-NPs efficacy was observed after 24 h. At 24 h, for *P. gingivalis* all tested concentrations of Dox-NPs attained above 98% bacterial death. In general, most effective dosage of Dox-NPs was found to be 10 mg/mL.

When testing Ca, Zn-doped or even undoped-NPs for *S. mutans* and *L. lactis*, bacterial viability was significantly affected in doses and time dependent manners. After 24 h, only those NPs contained 10 mg/mL were effective. Both bacteria were equally susceptible to Zn-NPs (68% cells reduction). When considering Ca-NPs or undoped-NPs, *L. lactis* was more susceptible (reduction values for Ca-NPs: 90%, for unloaded NPs: 70%) than *S. mutans* (reduction values for Ca-NPs: 60%, for unloaded NPs: 50%). 

Testing of NPs doped with Ca and Zn-doped NPs at 0.1 mg/mL against *P. gingivalis*, bacterial viability was significantly affected and bacterial death ranges between 55% to 27%. However, at the most effective concentration −10 mg/mL, bacterial reduction ranges were from 80% to 93%, without significant differences between both ion-doped NPs. *P. gingivalis* incubated with unloaded NPs attained low but dose and time-dependent percentages of bacterial survival reduced, from 34.3% to 7.2%.

## 3. Discussion

There are several in vitro testing models for the efficacy of antibacterial agents, which may involve single or multispecies bacteria. This microcosm model is the most clinically relevant, but attained results are often difficult to interpret as there is no way to control for the behavior of individual bacterial species. It is also difficult to decide which species are appropriate in each experiment and their relative amounts [3]. Using biofilm models is also challenging as the results may also be different on various materials surfaces with different chemistry and/or topography [30]. Therefore, when analyzing novel antibacterial agents planktonic monoculture tests are necessary to facilitate results interpretation. Further studies need to be conducted on clinical isolates and multi-species biofilms on different material surfaces or interfaces, which may express resistance trait against tested antibacterial effect.

*P. gingivalis* was selected for the present study as it is one of the most frequently detected anaerobic microorganisms in subgingival plaque samples from periodontal-endodontic combined lesions and necrotic pulp [6]. *S. mutans*, *S. gordonii*, *S. sobrinus* and *L. lactis* were used as are the most frequently detected microorganisms in cariogenic plaque [3]. *P. gingivalis* is a Gram-negative bacteria, *S. mutans*, *S. gordonii*, *S. sobrinus* and *L. lactis* are Gram positive. *P. gingivalis* has an asymmetric distribution of lipids at their cell walls, the outer face contains lipopolysaccharide (LPS), and the inner face has phospholipids [8]. *S. gordonii*, *S. sobrinus S. mutans* and *L. lactis* also have LPS at their membranes, which exhibits anionic charge, as a result it may facilitate cationic groups to bond and exert antimicrobial activity [8]. This may be a reason for observing low antibacterial activity of tested non-loaded NPs, as they are also anionic (potential zeta is −41 ± 5 mV measured in water at pH = 7) [20], and will not be attracted to tested bacteria which posse a zeta potential of approximately −25 mV at pH = 7 [31].

Ca-NPs and Zn-NPs exerted antibacterial activity, at 10 mg/mL 80% to 93% bacterial reduction after 48 h, was encountered (Figure 1) as a possible result of liberated calcium and zinc from NPs. After 48 h, 0.9 and 0.02 µg per 10 mg of NPs of calcium and zinc are respectively released (Table 1). Cationic metals as calcium or zinc have been shown to be potent antimicrobials [24,25,27]. Calcium release from NPs was estimated to be 0.08 and 0.1 µg/mL (per mg of NPs) from 12 h up to 7 days, while zinc release was around 0.02 µg/mL (per mg of NPs) at the same time-points. Cummulative liberation of both ions is 30% for calcium and 0.3% for zinc after 48 h. It has been shown that lipopolysaccharides at the outer membrane of Gram-negative bacteria possess magnesium and calcium ions that bridge to negatively-charged phosphor-sugars [8]. Therefore, cationic elements may also displace these metal ions damaging the outer membrane, leading to cell death [8,26]. It has also been previously shown that zinc ions markedly enhanced the adhesion and accumulation of salivary and serum proteins on cells of *P. gingivalis* and inhibited their coaggregation when growing on biofilms [27].

Zinc has a known inhibitory effect on glycolysis and proteinase activity in many oral bacteria [27]. Zinc may affect *S. mutans* viability by inhibiting glycolysis [32]. Kinetic studies of the glucosyltransferases of *S. sobrinus* by Devulapalle and Mooser [33] showed that the Zn ion acts as a reversible, competitive inhibitor at the fructose subsite within the active site of the glucosyltransferase. This observation may well explain the reported dose-dependent effects of zinc on the tested bacteria. Even when the exact antibacterial mechanism of zinc has not been clearly identified yet, covalently or oxidatively induced damage has been claimed [32]. Zinc ions are considered useful for limiting the settlement/colonization of *P. gingivalis* in the gingival sulcus with the goal of preventing periodontal disease [27] and in the case of *S. mutans* preventing carious disease [32]. Zinc has long been known as a plaque-inhibiting compound and also can influence acid production by different microbes [32]. In addition, zinc is able to depolarize the membrane potential, it does not always cause the bacterial cell membrane to rupture and leak, but alters permeability that is closely related to the sensitivity of bacteria to ionic environment [30]. Ion homeostasis affects the proliferation, communication and metabolism of bacteria; then, zinc may sometimes produce an inhibitive instead of destructive effect against bacteria [30].

Dox-NPs exerted the highest antibacterial activity to all the tested concentrations (80% to 97% bacterial reduction after 24 h). Following our results, doxycycline was found to be released at sustained levels for over 28 days, with a significant burst effect at 24 h. It is liberated at concentrations high above to those considered effective against bacteria at any time point of the present study. For each mg of NPs 121, 106 and 46 µg/mL of doxycycline will be liberated at 12, 24 and 48 h, respectively. A burst effect with rapid doxycycline liberation was observed from 12 h until the first week of storage. After 7 days time-point, antibiotic release was maintained above 20 µg/mL (per mg of NPs). As bacterial susceptibility to doxycycline is obtained around 0.1 to 0.2 µg/mL [28], doxycycline is then liberated from NPs at concentrations high above to those considered effective against most of the tested bacteria. It was shown that doxycycline at a concentration between 0.5 and 1 µg/mL is bactericidal against different *Pg* strains [34], and between 0.1 and 6.0 µg/mL is effective against *Pg* and other putative periodontal pathogens [35,36]. It should be stressed that tested Dox-NPs after 28 days are able to liberate doxycycline concentrations above 6 µg/mL.

Doxycycline is a polar and amphoteric compound. Doxycycline as a salt (hyclate) is water soluble. Doxycycline is known to act against most bacteria by inhibiting the microbial protein synthesis that requires access into the cell wall and lipid solubility [37]. Doxycycline binds the ribosome to prevent ribonucleic acid synthesis by avoiding addition of more amino acid to the polypeptide [37]. Doxycycline is also known to provoke a potent and long-lasting inhibition of dentin matrix metalloproteinases [29] that are related to chronic inflammation processes and abscesses at apical level [10]. It may explain how long-term administration of a sub-antimicrobial dose of doxycycline, to dogs with periodontitis, is regarded as an effective treatment for periodontal inflammation, even when it does not induce antimicrobial effects [38]. It is also important to note that MMPs inhibition may also prevent collagen degradation at the resin bonded dentin interface [15]. It will also probably reduce secondary caries formation, as MMPs activity is augmented at caries affected dentin [39]. 

The reported doxycycline liberation data are high and sustained, if compared to the release profile of other previously proposed compounds as a cellulose-acetate-loaded doxycycline formulation studied by Tonetti et al. [40]. Kim et al. [28] introduced a biodegradable doxycycline gel and reported a mean local concentration of 20 mg/mL, after 15 min; values that were lowered to 577 µg/mL after 3 days and to 16 µg/mL after 12 days. Deasy et al. [41] used tetracycline hydrochloride in poly(hydroxybutyric acid) as a biodegradable polymer matrix and showed sustained release just over 4 to 5 days, with a significant burst effect at 24 h. Previously introduced materials are then able to liberate doxycycline at higher concentrations, but in shorter periods of time, denoting accentuated burst effects. 

In general, tested NPs had little effect on the growth of (*S. sobrinus* and *S. gordonii*) and specially Dox-NPs after 24 h, which greatly affected *P. gingivalis*, *S. mutans* and *L. lactis* survival rates (at least at the evaluated time points and concentrations). Recent results on advanced caries lesions in young human teeth, using bacterial sequence analysis methods are consistent and indicate that *S. gordonii* diminishes greatly in caries-associated plaque biofilm, while *S. mutans* persists [42]. It means that NPs may selectively inhibit cariogenic and periodontal bacteria, while leaving commensal microbes. However, it should be assayed in properly designed multibacteria biofilms models in future studies. It is to be noted that the tested NPs are biocompatible against human fibroblasts [23], and the application of antibacterials is crucial if regenerative/revascularization processes are performed for the endodontic treatment [43], these NPs may be an interesting tool. 

Two important limitations are recognized for the present Dox-NPs: (1) antibiotics may produce bacterial strain resistance, which is a current global concern; therefore, further research is needed. (2) The bacteria grow in biofilms, and are known to be more resistant to antimicrobial treatment than the planktonic cultures used for the present antimicrobial susceptibility testing [44]. Then, it is imperative to include the biofilm mode of growth of bacteria when testing treatments for bonded dentin interfaces, endodontic and periapical diseases. But these tests are difficult to control, in terms of knowing specifically how bacteria are involved in the process [3]. It will not be possible to ascertain if a specific toxicity of NPs against individual bacteria is being produced [44], or just a biofilm disruption interfering with first colonizers bacteria attachment to dentin. 

It may be concluded after this in vitro study that experimental NPs loaded with zinc, calcium or doxycycline are effective to eradicate tested oral bacteria. For clinical applications, using these NPs at the resin-bonded interface as cavity liners may be recommended. As it was shown before, that NPs do not affect bond efficacy and improve dentin remineralization [17,21,22]. The same beneficial effect may be found if NPs are used in endodontics, before resin sealant cement application. However, as recognized in the study limitations, further investigations into antibacterial effects through biofilm models of multiple bacterial species should be implemented. 

## 4. Material and Methods

*1. Preparation of Nanoparticles (NPs):* PolymP-*n* Active NPs were acquired (NanoMyP, Granada, Spain). Particles are fabricated trough polymerization precipitation. Main components of NPs are 2-hydroxyethyl methacrylate, ethylene glycol dimethacrylate and methacrylic acid; these compounds are the backbone monomer, the cross-linker and the functional monomer respectively.

Calcium-loaded NPs (Ca-NPs) and Zinc-loaded NPs (Zn-NPs) were produced. Zinc and calcium complexation was obtained immersing 30 mg of NPs during 3 days, under continuous shaking in aqueous solutions of ZnCl_2_ or CaCl_2_, at room temperature. Fifteen mL of the solutions containing zinc or calcium at 40 ppm were employed (pH 6.5). Then, the adsorption equilibrium of metal ions was reached [23]. To separate the NPs from the supernatant, the suspensions were centrifuged. 0.96 ± 0.04 µg Ca/mg NPs and 2.15 ± 0.05 µg Zn/mg NPs were the attained ion complexation values [23]. NPs loaded with doxycycline hyclate were also produced. An 18-mL aqueous solution, with 40 mg/mL of doxycycline hyclate (Sigma-Aldrich, Darmstadt, Germany) was prepared, and 30 mg of NPs were immersed in the solution for 4 h, under continuous shaking. Then, to separate NPs from the supernatant the suspensions were centrifuged. Following groups were tested: (1) NPs (NPs), (2) NPs loaded with Ca (Ca-NPs), (3) NPs loaded with Zn (Zn-NPs), and (4) NPs with immobilized doxycycline hyclate (Dox-NPs).

*2. Loading efficacy and release of doxycycline from NPs:* For loading efficacy 18 mL of 40 mg/mL aqueous solution of doxycycline hyclate was prepared and the amount of doxycycline in the initial aqueous solution was assessed in triplicate samples of 100 μL and recorded as initial doxycycline concentration (1333 µg Dox/mL). Three different samples containing 1 mg of NPs and 0.6 mL of the doxycycline solution were incubated for 4 h, under continuous shaking. Then, the suspensions were centrifuged and the particles were separated from the supernatant, 100 μL of each supernatant was analyzed for final doxycycline concentration. Final doxycycline concentration was subtracted from initial values to calculate loading efficacy [45]. To ascertain for doxycycline liberation, 30 mg of doxycycline loaded-NPs were suspended in 3 mL of phosphate buffer saline (PBS, pH 7.4, Fisher Scientific SL, Madrid, Spain), three different eppendorf tubes containing 1 mL of the Dox-NPs suspension were stored at 37 °C. After 12 h, suspensions were centrifuged and the particles were separated from the supernatant. An aliquot (0.1 mL) of each supernatant was analyzed for doxycycline concentration. NPs were washed and 1 mL of fresh PBS was used to resuspend the NPs at 10 mg/mL until the next supernatant collection. Seven different time-points were tested: 12, 24, 48 h, 7, 14, 21 and 28 days. Supernatans were stored at −20 °C until doxycycline concentration measuring [45]. The amount of doxycycline was assayed by high performance liquid chromatography (HPLC) (Waters Alliance 2690, Waters Corporation, Milford, MA, USA) equipped with a UV-Vis detector. A binary mobile phase consisting of solvent systems A and B was used in an isocratic elution with 80:20 A:B. Mobile phase A was 50 mM KHPO_4_ in distilled H_2_0 and mobile phase B was 100% acetonitrile. The HPLC flow rate was 1.0 mL/min and the total run time was 10 min. The retention time was 4.85 min. The concentration of doxycycline was calculated based on a standard curve of known levels of doxycycline at 273 nm [45].

*3. Calcium and zinc liberation from NPs:* 150 mg of zinc and 150 mg of calcium loaded-NPs were suspended in 15 mL of deionized water. Three different eppendorf tubes containing 5 mL of the Ca-NPs suspensions and other 3 with Zn-NPs were stored at 37 °C. After 12 h, suspensions were centrifuged and the particles separated from the supernatant; 5 mL of each supernatant was analyzed for calcium and zinc concentration. NPs were washed and 5 mL of fresh deionized water was used to resuspend the NPs at 10 mg/mL until the next supernatant collection. Seven different time points were tested: 12 h, 24 h, 48 h, 7 d, 14 d, 21 d and 28 d. Supernatans were stored at −20 °C until testing. Calcium and zinc concentrations were analyzed through an inductively coupled plasma (ICP) optical emission spectrometer (ICP-OES Optima 8300, Perkin-Elmer, MA, USA) [23]. 

*4. Bacteria: P. gingivalis* 33,277, *S. mutans* 700,610, *S. sobrinus* 33,478, *S. gordonii* 10,558 and *L. lactis* 12,315 were obtained from ATCC (Bethesda, MD, USA). The anaerobic organism, *Pg* was grown in Tryptic Soy broth (TSB) supplemented with yeast extract (5 g/L), Hemin (5 mg/L), Menadione (1 mg/L), for 72 h. Strict anaerobic conditions were employed, *Thermo Scientific* Oxoid *AnaeroGen* (Thermo Fisher Scientific, Waltham, MA, USA) was used in an anaerobic jar, which provides 7–15% CO_2_ and <0.1% O_2_. The remaining test bacteria were grown in TSB for 24 h at 37 °C. The bacterial cells were harvested by centrifugation and re-suspended in the same growth media. The number of bacteria per mL was determined by measuring the optical density at 600 nm and adjusting it to a standard bacterial suspension of 1 × 10^7^ CFU/mL [46].

*5. MTT assay:* The NPs were suspended in PBS at three different concentrations (10 mg/mL, 1 mg/mL and 0.1 mg/mL). NPs were placed into Eppendorf tubes with bacterial broths (1 × 10^7^ CFU/mL for each 0.45 mL of NPs suspensions) and incubated for 3, 12 and 24 h at 37 °C. Sterile pipetting was used throughout the study. Susceptibility testing of *P. gingivalis* was conducted in an anaerobic jar as described above. At the end of each incubation period, the effect of the NPs on bacteria was evaluated by the ability of viable bacteria to cleave the tetrazolium salt (3-[4,5-dimethylthiazol-2-yl]-2,5-diphenyl tetrazolium bromide) (MTT) to a formazan dye (Sigma-Aldrich, Darmstadt, Germany). 96-well flat-bottom microtiter plates were used to place the suspensions in. The plates were incubated for 4 h at 37 °C, after the MTT labeling agent addition to each culture well. Then, the solubilizing agent that was provided by the manufacturer was added and an overnight incubation at room temperature was performed. An enzyme-linked immunosorbent assay (ELISA) reader (Spectrostar Nano, BMG Labtech, Cary, NC, USA) was employed, the purple formazan color that was produced from the MTT by viable cells, was read (560 nm) [46]. Assays were performed with three determinants, and experiments were performed in triplicate. Data expressed as mean ± standard deviation were analyzed by one-way analysis of variance (ANOVA) and the post hoc comparisons Scheffe’s F tests, at *p* < 0.05, using SPSS Statistic 20.

## Figures and Tables

**Table 1:** Mean and standard deviation (SD) of Ca^2+^, Zn^2+^ and doxycycline liberation in µg. Cumulative liberation (CL) was expressed in percentages. Values are obtained per 10 mg of NPs, at each time point.

Time	Ca^2+^ (µg)	Ca^2+^ CL (%)	Zn^2+^ (µg)	Zn^2+^ CL (%)	Doxycycline (µg)	Doxycycline CL (%)
12 h	1.006 (0.002)	11	0.025 (0.001)	0.1	1211.29 (166.32)	30
24 h	1.007 (0.001)	21	0.025 (0.001)	0.2	1065.98 (146.15)	57
48 h	0.909 (0.003)	30	0.023 (0.002)	0.3	458.08 (63.5)	68
7 days	0.856 (0.001)	39	0.021 (0.001)	0.4	210.81 (28.33)	74
21 days	2.082 (0.05)	61	0.024 (0.002)	0.5	81.85 (10.97)	78
28 days	2.031 (0.02)	82	0.044 (0.005)	0.8	63.23 (9.01)	80
